# The acute effects of cannabidiol on the neural correlates of reward anticipation and feedback in healthy volunteers

**DOI:** 10.1177/0269881120944148

**Published:** 2020-08-05

**Authors:** Will Lawn, James Hill, Chandni Hindocha, Jocelyn Yim, Yumeya Yamamori, Gus Jones, Hannah Walker, Sebastian F Green, Matthew B Wall, Oliver D Howes, H Valerie Curran, Tom P Freeman, Michael AP Bloomfield

**Affiliations:** 1 Clinical Psychopharmacology Unit, University College London, London, UK; 2 Translational Psychiatry Research Group, University College London, London, UK; 3 NIHR University College London Hospitals Biomedical Research Centre, University College Hospital, London, UK; 4 Institute of Cognitive Neuroscience, University College London, London, UK; 5 Department of Neuroscience, Physiology and Pharmacology, University College London, London, UK; 6 Invicro London, Hammersmith Hospital, London, UK; 7 Psychiatric Imaging Group, Imperial College London, London, UK; 8 Addiction and Mental Health Group (AIM), University of Bath, Bath, UK; 9 National Addiction Centre, Institute of Psychiatry, Psychology and Neuroscience, London, UK; 10 The Traumatic Stress Clinic, St Pancras Hospital, London, UK; 11 National Hospital for Neurology and Neurosurgery, London, UK

**Keywords:** Cannabidiol, reward, functional magnetic resonance imaging, motivation, anticipation, feedback, cannabis, marijuana

## Abstract

**Background:**

Cannabidiol has potential therapeutic benefits for people with psychiatric disorders characterised by reward function impairment. There is existing evidence that cannabidiol may influence some aspects of reward processing. However, it is unknown whether cannabidiol acutely affects brain function underpinning reward anticipation and feedback.

**Hypotheses:**

We predicted that cannabidiol would augment brain activity associated with reward anticipation and feedback.

**Methods:**

We administered a single 600 mg oral dose of cannabidiol and matched placebo to 23 healthy participants in a double-blind, placebo-controlled, repeated-measures design. We employed the monetary incentive delay task during functional magnetic resonance imaging to assay the neural correlates of reward anticipation and feedback. We conducted whole brain analyses and region-of-interest analyses in pre-specified reward-related brain regions.

**Results:**

The monetary incentive delay task elicited expected brain activity during reward anticipation and feedback, including in the insula, caudate, nucleus accumbens, anterior cingulate and orbitofrontal cortex. However, across the whole brain, we did not find any evidence that cannabidiol altered reward-related brain activity. Moreover, our Bayesian analyses showed that activity in our regions-of-interest was similar following cannabidiol and placebo. Additionally, our behavioural measures of motivation for reward did not show a significant difference between cannabidiol and placebo.

**Discussion:**

Cannabidiol did not acutely affect the neural correlates of reward anticipation and feedback in healthy participants. Future research should explore the effects of cannabidiol on different components of reward processing, employ different doses and administration regimens, and test its reward-related effects in people with psychiatric disorders.

## Introduction

Reward processing refers to the neural, psychological and behavioural processes that underpin the seeking and consumption of rewards (Berridge et al., 2009). The human brain reward system is made up of key regions such as the ventral tegmental area (VTA), ventral and dorsal striatum, anterior cingulate cortex, orbitofrontal cortex, ventral pallidum, amygdala, insula, thalamus and parahippocampal regions (Haber and Knutson, 2010; Knutson and Greer, 2008). Fronto-striatal loops pass reward-related information from the prefrontal cortex to subcortical regions and back again, such that organisms can orient attention to, be motivated for, and consume rewards (Haber and Knutson, 2010).

Reward processing is perturbed in a variety of psychiatric disorders, including depression (Eshel and Roiser, 2010; Knutson et al., 2008; Whitton et al., 2015), addiction (Balodis and Potenza, 2015; Goldstein and Volkow, 2011) and schizophrenia (Gold et al., 2008; Juckel et al., 2006; Strauss et al., 2013). Dysfunctional reward processing therefore represents an important transdiagnostic neurocognitive mechanism which may contribute to the emergence of various psychiatric disorders (Husain and Roiser, 2018; Insel, 2010; Whitton et al., 2015). Hence, the reward circuit is a potential target for novel psychiatric drug treatments. Successful manipulation of the reward system could lead to the amelioration of impaired reward learning, motivation and pleasure, observed across various clinical diagnoses.

The endocannabinoid system plays an important role in modulation of the brain’s reward processes (Bloomfield et al., 2016; Parsons and Hurd, 2015; Solinas et al., 2009). Cannabinoid type-1 receptors are expressed at a moderate level at the origin of the mesolimbic dopamine pathway, the VTA, and at a higher level at the terminal region, the nucleus accumbens (NAcc) (Curran et al., 2016; Solinas et al., 2009).

Cannabidiol (CBD) is the second most abundant cannabinoid in the cannabis plant (Pertwee, 2008) and at typical doses CBD is non-intoxicating (Haney et al., 2016; Hindocha et al., 2015; Lawn et al., 2016; Martin-Santos et al., 2012). CBD has therapeutic potential in a variety of psychiatric disorders (Freeman et al., 2019; Khan et al., 2020). Preclinical research has demonstrated that CBD administration can affect reward-related behaviours, particularly reducing drug-seeking behaviour (Hay et al., 2018; Katsidoni et al., 2013; Parker et al., 2004; Ren et al., 2009; Schier et al., 2014; Viudez-Martínez et al., 2018). Speculatively, CBD could ameliorate addictive behaviour by enhancing the sensitivity of the reward system to natural rewards, such that pharmacological rewards are less desired. The effects of CBD on the mesolimbic dopamine system are, however, equivocal (Renard et al., 2017).

Human research has shown that CBD can acutely alter neural, behavioural and psychological processes relating to reward, including effort sensitivity (Lawn et al., 2016), attentional bias to drug pictures (Hindocha et al., 2018; Morgan et al., 2010), drug consumption (Freeman et al., 2020; Morgan et al., 2013), neural response to music reward (Freeman et al., 2018) and levels of stress-induced social anxiety (Bergamaschi et al., 2011; Zuardi et al., 1993), without producing reinforcing or unpleasant side-effects (Haney et al., 2016). However, it is not known if CBD specifically acts on the human brain’s reward circuitry, or acts by another mechanism. Furthermore, if CBD does act on the reward system, its effects on reward anticipation and reward feedback have not been parsed.

The monetary incentive delay (MID) task is a well-validated functional magnetic resonance imaging (fMRI) task which, through its structure, allows for investigation of the neural correlates of reward anticipation and reward feedback (Balodis and Potenza, 2015; Knutson et al., 2001). Meta-analyses of MID task results show reward anticipation and feedback recruit overlapping and distinct regions (Knutson and Greer, 2008; Oldham et al., 2018). Both processes activate striatal regions, while reward anticipation activates the thalamus and insula, and reward feedback preferentially activates prefrontal cortex areas. Importantly, neural activity during reward anticipation in the ventral striatum correlates with dopamine release in the same region (Schott et al., 2008), demonstrating the task engages the mesolimbic dopamine system.

CBD seemingly has opposite effects to the primary intoxicating cannabinoid found in cannabis, delta-9-tetrahydracannabinol (THC), on both brain and behaviour (Bhattacharyya et al., 2010; Bloomfield et al., 2016; Englund et al., 2013). CBD enhanced striatal activation during a verbal memory task, while THC dampened striatal activity (Bhattacharyya et al., 2010). In the MID task, acute THC administration has been shown to attenuate the widespread neural response to reward feedback (van Hell et al., 2012) and attenuate the neural response in the NAcc during reward anticipation in people with nicotine dependence (Jansma et al., 2013). Therefore, one might expect CBD to do the opposite: augment neural response to reward anticipation and feedback. Furthermore, a pro-reward function action could underlie CBD’s putative anti-addiction, anti-depressant and anxiolytic effects.

In summary, the endocannabinoid system plays an important role in the brain’s reward circuitry and both preclinical and human research has demonstrated that CBD can modulate reward-related behaviours. However, previous human studies have tended to investigate CBD’s impact alongside THC. Moreover, they have focused on psychiatric symptom-based measures, rather than precise components of reward processing, such as anticipatory and consummatory reward processes which are indexed by the well-validated MID task. No study has examined the specific, isolated effect of CBD on the human brain during reward processing. Based on its opposing effects to THC and its ostensibly therapeutic effects in disorders characterised by reward dysfunction, we predicted that CBD would augment the neural response to reward anticipation and feedback.

## Methods

### Design and participants

The study used a double-blind, randomised, placebo-controlled, repeated-measures design to compare the effects of oral CBD 600 mg with matched placebo (PBO). Drug order was balanced and randomised. Drug order was completely concealed from participants and concealed from experimenters until data collection, entry and analysis had been completed.

We tested 28 healthy participants. Four participants did not complete both sessions, so they were excluded. Furthermore, one participant did not complete the MID task correctly, so they were excluded. That left 23 participants in our analysis.

Participants were recruited through public advertisement. Inclusion criteria were: (a) age 18–70 years; (b) right-handed; and (c) fluent in English. Exclusion criteria were: (a) positive urine screen for recreational drug use (Alere Toxicology UC-10A; amphetamines, barbiturates, benzodiazepines, cocaine, methamphetamine, morphine, methadone, phencyclidine, tricyclic antidepressants, THC); (b) recent (within the past six months) use of any psychotropic (recreational or medical) drug, including cannabis; (c) positive breath test for alcohol; (d) carbon monoxide ⩾5 parts per million (ppm); (e) problematic alcohol use, as defined by a score ⩾8 on the Alcohol Use Disorder Identification Test (AUDIT) (Saunders et al., 1993); (f) more than 10 lifetime uses of cannabis or CBD; (g) more than five lifetime uses of any other recreational drug; (h) nicotine-dependent, as defined by a score >3 on the Fagerstrom Test for Nicotine Dependence (FTND: Heatherton et al., 1991); (i) current or past mental or physical health issues or learning impairments, based on an adapted version of the Diagnostic and Statistical Manual of Mental Disorders-IV (DSM-IV) Structured Clinical Interview (SCID) (Gibbon and Spitzer, 1997); (j) positive reading on urine pregnancy test; (k) breast-feeding; (l) known allergies or aversions to CBD, microcrystalline cellulose, gelatine or lactose; (m) colour blindness; (n) magnetic resonance imaging (MRI) contraindications; and (o) current use of psychiatric medications.

Participants were reimbursed £10/h for their time. This study was approved by the University College London (UCL) Ethics Committee (Project number: 3325/002), and all participants provided written informed consent.

### Assessments

#### The MID task (Figure 1)

**Figure 1.**
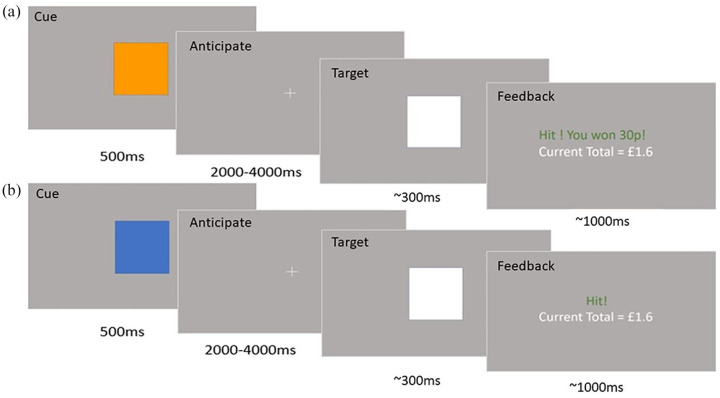
The monetary incentive delay (MID) task. (a) A ‘win hit’ trial example. An orange square is presented which signals that it is a ‘win’ trial, in which the participant has the opportunity to win 30p. Then, there is an ‘anticipation’ phase (a blank screen with a crosshair), while the participant anticipates the onset of the target. Then, there is a target, which if responded to in a short amount of time (which varies based on participant performance, starting at 300 ms) leads to money being won. Then, feedback is given; in this case feedback is positive because the participant hit within the target time on a win trial. Subsequently, there is an inter-trial interval (a blank screen) for 2.2–10.2 s. (b) A ‘neutral hit’ trial example. A blue square is shown instead of an orange square, signalling no money is available. The rest of the trial is the same but when the participant hits the target in time, monetary reward feedback is not subsequently presented.

The MID task is a well-validated task that allows measurement of neural activity during reward anticipation and reward feedback using fMRI. We used an adapted version of the original (Knutson et al., 2000).

In our version of the task, a cue (a square) is first presented for 500 ms, which signals whether the trial is a win trial (if the square is orange) or a neutral trial (if the square is blue). On a win trial, the participant has the opportunity to win 30p if they respond to a subsequent target in time. On a neutral trial, the participant cannot win or lose any money, but they are asked to respond to the subsequent target as quickly as they can anyway. Following the cue, there is a blank screen, the anticipation phase, for 2–4 s in which the participant waits for the target. Subsequently, the target (a white square) is presented and the participant must respond to it as quickly as they can by pressing a button with their thumb on their right hand. Initially, participants must respond to the target within 300 ms in order to get a ‘hit’. However, following a successful ‘hit’, the next trial’s target must be responded to within a time that is 16.67 ms shorter than the previous trial in order to get another ‘hit’. Following a ‘miss’, the next trial’s target must be responded to within a time that is 16.67 ms longer than the previous trial in order to get a ‘hit’. This is to calibrate the participant’s ‘hit’ success to roughly 50% of the time.

Following the target, feedback is presented for roughly 1000 ms (although this changes on a trial-by-trial basis along with changes in target duration). If it is a ‘win’ trial and the participant gets a ‘hit’, then the participant wins 30p and is told ‘Hit. You win 30p’. If it is a ‘win’ trial and the participant gets a ‘miss’, then the participant does not win money and is told ‘Miss’. If it is a ‘neutral’ trial and the participant gets a ‘hit’, then the participant does not win money and is told ‘Hit’. If it is a ‘neutral’ trial and the participant gets a ‘miss’, then the participant does not win money and is told ‘Miss’. The current total won is always displayed on the feedback screen. Following the feedback, there is an inter-trial interval (ITI) between 1.2–9.2 s when a blank screen is shown.

There were 48 trials in total, of which 24 were neutral trials in which no money could be earned and 24 were win trials in which money could be earned. The order of win trials was fixed, so that win trials did not appear consecutively. Each win trial provided the opportunity to win 30p; this amount did not vary, as in some previous MID task versions (Knutson et al., 2008). There were also no loss trials. The task lasted for 12 min.

The MID task produces measures of brain activity associated with reward anticipation and reward feedback. It also produces behavioural measures of: (1) mean reaction time (RT) to respond to the target on successful ‘win’ and ‘neutral’ trials and (2) the proportion of ‘hits’ on ‘win’ and ‘neutral’ trials.

#### Demographics

We recorded participants’ age, sex, weight and body mass index (BMI).

#### Beck Depression Inventory (BDI)

The BDI is a self-reported scale of depression severity which consists of 21 items (Beck et al. 1996). This was used to measure the participants’ depressive symptomatology over the two weeks preceding the first study visit. Higher scores reflect a higher severity of depression.

#### AUDIT

The AUDIT is a self-reported scale which screens for problematic alcohol use and consists of 10 items (Saunders et al., 1993). Scores range from 0–40, with higher scores reflecting more severe problematic alcohol use. A score of eight or more is considered hazardous.

#### FTND

The FTND is a self-reported scale of nicotine dependence consisting of six items (Heatherton et al., 1991). Total scores range from 0–10, with higher scores reflecting higher nicotine dependence.

#### Wechsler Test for Adult Reading (WTAR)

The WTAR is a test of reading ability which is a proxy of verbal intelligence (Ginsberg et al., 2003). It includes 50 words that must be read aloud and pronounced correctly.

#### Plasma CBD levels

Blood samples were collected using EDTA vacutainers and centrifuged immediately. Plasma samples were stored at −80^o^C prior to analysis. CBD concentrations were determined using gas chromatography mass spectroscopy (GC/MS) with a lower limit of quantification of 0.5 mg/mL.

### Drug administration

Participants were administered a single dose of 600 mg oral CBD (pure synthetic (-)-CBD, STI Pharmaceuticals, Essex, England) or matched PBO (lactose powder) in identical, opaque capsules on each testing session. The CBD was formulated in 50 mg capsules. Participants swallowed all 12 capsules at their own pace under invigilation of the experimenter. The amount of 600 mg was chosen as it produces an increase in plasma concentrations after acute administration (Babalonis et al., 2017; Englund et al., 2013), is well tolerated in humans (Grotenhermen et al., 2017), produces a significant anxiolytic effect (Bergamaschi et al., 2011), produces opposing effects to THC on the striatum as assessed by fMRI (Bhattacharyya et al., 2010) and elicits anti-psychotic like effects in combination with THC (Bhattacharyya et al., 2015).

### Procedure

Participants completed a screening on the telephone during which initial eligibility criteria (drug use, FTND, AUDIT, MRI contraindications, allergies, medical information and handedness) were assessed and basic participant details were recorded. Participants that appeared eligible on the phone were invited to attend experimental sessions. Participants were asked to fast from midnight the day before both sessions, and refrain from smoking tobacco and consuming alcohol for 24 h before the start of the sessions. Upon arrival, participants underwent urine tests to verify they were not pregnant (if female) and they had not recently taken recreational drugs. They also completed breath tests for alcohol and carbon monoxide.

Eligible participants then completed two seven-hour experimental sessions, when they received CBD or PBO on the first session, and the other drug condition on the second session. Experimental sessions were separated by a minimum seven-day wash-out period (>4 times the elimination half-life) to minimise carryover effects of CBD (Consroe et al., 1991). The BDI and WTAR were completed immediately after drug administration on the second session.

Previous research suggests that CBD reaches the peak level of plasma concentration after approximately 2.5 h (Babalonis et al., 2017). Therefore, 2.5 h after drug administration, participants underwent MRI scanning for 1.5 h to complete the MID task, as well as other tasks and scans, which will be reported elsewhere. Participants’ blood samples were taken straight after the scan finished, which was approximately 4 h and 15 min after drug administration. After a standardised lunch provided by the experimenter, participants completed a series of questionnaires and computer tasks, results of which will be reported elsewhere.

### Power calculation

A power calculation was conducted using G*Power (version 3.1.9.2). This showed that a sample size of 20 would have 81% power to detect a significant (*p*<0.05, two-tailed) difference between CBD and PBO with a moderate or greater effect size of *d*=0.5. This effect size was based on the previous finding of the difference in the attentional bias toward cigarette cues between 800 mg CBD versus PBO in nicotine-dependent users (Hindocha et al., 2018). We then recruited extra participants to account for expected participant dropout and exclusions.

### MRI data acquisition

MRI data was collected using a 3-Tesla Siemens Verio MRI Scanner at the Robert Steiner MR unit at Hammersmith Hospital, London. Functional imaging used a multiband (acceleration factor=2) gradient-echo T2*-weighted echo-planar imaging (EPI) sequence with 42 slices per volume (Repetition time [﻿TR]=2400 ms; Time to Echo [﻿TE]=30 ms; in-plane matrix=64×64; 3 mm isotropic voxels; flip angle=62°; bandwidth=1594 Hz/pixel; 304 volumes; a slice thickness of 3 mm; field of view=192 × 192 mm). The phase encoding direction was from anterior to posterior. Echo spacing was 0.71 ms. There were three dummy scans at the beginning of the scan, which were not included in in our dataset. For structural acquisition, a T1-weighted structural volume was acquired for all participants using a magnetisation prepared rapid gradient echo (MPRAGE) scan (TR=2300 ms; TE=2.28 ms, TI=900 ms, flip angle=9°, field of view= 256 mm, image matrix=256 with 1 mm isotropic voxels; bandwidth=200 Hz/pixel).

### fMRI data analyses

Image pre-processing and analysis were performed using FSL’s fMRI Expert Analysis Tool (FEAT) (FMRIB Software Library v6.0, Analysis Group, FMRIB, Oxford, UK) (Jenkinson et al., 2012). Data were pre-processed before being subject to first and second-level analyses.

#### Pre-processing

FSL’s brain extraction tool (BET) was used to strip the brain from the skull. The FMRIB Automated Segmentation Tool was used to separate out grey matter, white matter and cerebrospinal fluid. Functional images were realigned to the middle volume using FSL’s MCFLIRT procedure, in order to correct for head motion. Subsequently, the functional images were co-registered to the individual participant’s structural image and normalised to the Montreal Neurological Institute (MNI-152) template using FEAT’s non-linear transformation procedure with a 10 mm warp resolution. An isotropic 6 mm full-width at half-maximum Gaussian kernel (i.e. twice the voxel size) was then applied to spatially smooth images. A high-pass filter (100 s cut-off) was applied to remove low-frequency noise. Images were visually inspected to ensure that the pre-processing had worked correctly.

T_1_-weighted structural images were also skull-stripped with FSL’s BET and normalised to the MNI-152 template.

#### First level analyses

Timestamps and durations for each event (cue, anticipate, target, feedback, ITI) in the MID task were extracted from the task output files using scripts written in Matlab (Mathworks Inc., USA). A general linear model was created with the following explanatory variables (i.e. regressors): (a) reward anticipation (i.e. anticipate-win), (b) no reward anticipation (i.e. anticipate-neutral), (c) reward feedback on a successful win trial (i.e. feedback-win-hit), (d) no reward feedback on an unsuccessful win trial (i.e. feedback-win-miss), (e) no reward feedback on a successful neutral trial (i.e. feedback-neutral-hit), (f) no reward feedback on an unsuccessful neutral trial (i.e. feedback-neutral-miss). Each event was modelled with a boxcar function with the event’s duration convolved with the canonical haemodynamic response function, using the gamma function. Extended motion parameters and temporal derivatives were included as additional regressors-of-no-interest.

The following contrasts were then calculated:

1. ‘Reward anticipation’: anticipate-win > anticipate-neutral.
2. ‘Reward feedback’: feedback-win-hit > feedback-neutral-hit.

#### Second level analyses

##### Whole brain analysis

The second-level fMRI data analysis was also performed with FSL’s FEAT pipeline (Jenkinson et al., 2012), using a random effects analysis with FMRIB’s Local Analysis of Mixed Effects (FLAME). We analysed the two contrasts specified above at the second level. We used clusterwise correction, with a cluster-defining threshold of *z*=2.3 and an alpha value of 0.05.

We conducted one-sample *t*-tests for both contrasts, collapsing across both drug conditions, to investigate the overall effect of the task (reward anticipation and reward feedback) on brain activity. Secondly, we conducted paired *t*-tests for both contrasts to investigate the differences, in both directions, between CBD and PBO.

##### Region of interest (ROI) analyses

ROIs were pre-specified based on a meta-analysis of MID fMRI results for significantly activated regions for reward anticipation and feedback (Knutson and Greer, 2008). There were eight ROIs for anticipation and seven ROIs for feedback, as shown in Table 1. The Talairach coordinates from Knutson and Greer (2008) were converted to MNI coordinates using the mni2tal MATLAB function created by the University of Cambridge Medical Research Council Cognition and Brain Sciences Unit (http://imaging.mrc-cbu.cam.ac.uk/imaging/MniTalairach). We used these coordinates as the centres for our spherical ROIs, with radii of 5 mm. The ROIs were created using FSLeyes and fslmaths functions. We then extracted average unstandardised beta values (with arbitrary units) from these regions for the two contrasts described above.

**Table 1.** Montreal Neurological Institute (MNI) coordinates for the centres of our regions of interest (ROIs) for the anticipate and feedback contrasts based on Knutson and Greer (2008).

Atlas	MNI		
Region	*x*	*y*	*z*
**Reward anticipation contrast**			
L medial frontal gyrus	0	–4.5	52.0
R insula	32.3	18.5	1.0
R NAcc	10.1	8.1	2.6
L NAcc	–12.1	10.4	–1.8
R thalamus	4.0	–10.9	12.5
L thalamus I	–6.1	–23.0	5.3
L thalamus II	–2.0	–23.0	9.7
L culmen	0	–61.5	–10.7
**Reward feedback contrast**			
R subcallosal gyrus	8.1	2.5	–9.4
L parahippocampal gyrus	–18.2	–26.6	–6.3
R parahippocampal gyrus	22.2	–22.4	–6.1
R caudate	8.1	16.4	3.0
R NAcc	12.1	10.6	–6.5
L NAcc	–8.1	6.4	–4.4
L amygdala	–16.2	–.15	–12.0

L: left; NAcc: nucleus accumbens; R: right.

L Thalamus I is further to the left and is more inferior than L Thalamus II.

We then ran one-sample *t*-tests (against a score of zero) to test whether the task elicited the expected anticipation and feedback activation in the hypothesised regions. Subsequently, we ran paired *t*-tests for an effect of drug (CBD vs PBO) on the activation in these anticipation and feedback ROIs. We reduced the alpha value to 0.006 to account for the multiple tests (i.e. ROIs) within each contrast.

We examined the extracted beta values for normality by visually inspecting histograms of the data, checking for kurtosis and skewness values >1, using Kolmogrov-Smirnov tests and looking for outliers as shown by SPSS’s box and whisker plots. Across all regions, for both CBD and PBO and for both reward anticipation and feedback, the data were normally distributed, so data were left unchanged.

In order to gain further support for either the null or alternative hypothesis for the effects of CBD on brain activity during reward anticipation and feedback, we also calculated scaled Jeffreys-Zellner-Siow (JZS) Bayes factors using an online calculator (http://pcl.missouri.edu/bayesfactor) (Buckingham et al., 2016; Lawn et al., 2018). We used a scaled-information prior of *r=*1, which is the default value recommended (Rouder et al., 2009). For this analysis, a Bayes factor of >3 provides support for the null hypothesis (i.e. no difference in activation between CBD and PBO).

We conducted Pearson correlations between participant CBD plasma levels and their extracted beta values for each anticipate and feedback ROI, when they were on the CBD condition. We reduced the alpha value to 0.006 to account for multiple tests (i.e. ROIs) within each contrast.

### Behavioural analyses

We conducted a Wilcoxon signed-rank test on the plasma CBD levels for CBD compared with PBO.

We conducted 2×2 repeated-measures analyses of variance (ANOVAs) for RT and the proportion of hits, with within-subjects factors of drug (CBD, PBO) and trial-type (win, neutral).

## Results

### Demographics

Of the 23 participants included in the analysis, there were 12 women and 11 men, with mean age 23.74 years (standard deviation (SD)=4.2, range: 19–36). Participants’ depression (BDI mean=2.2, SD=4.9, range: 0–11) and problematic alcohol use (AUDIT mean=2.2, SD=2.8, range: 0–7) levels were low. Participants had a mean WTAR raw score of 40.5 (SD=4.9, range: 33–49) and a mean BMI of 22.4 kg/m^2^ (SD=3.5, range: 17.6–35.4).

### Plasma CBD levels

Plasma CBD levels were higher on CBD (median=6.01 ng/mL, interquartile range=4.89) than PBO (median=0, interquartile range=0) (*z*=3.296, *p*=0.001).

### MID behavioural results

For RT, there were main effects of drug (*F*_1,22=_6.286, *p*=0.020) and trial-type (*F*_1,22=_15.841, *p*=0.001), but there was not a significant interaction. Participants were faster to respond on win trials (mean=0.241 s, SD=0.023) compared to neutral trials (mean=0.247 s, SD=0.024). Participants were faster, overall, to respond under PBO (mean=0.241 s, SD=0.024) compared to CBD (mean=0.247 s, SD=0.024).

For proportion hit, there was a main effect of trial-type (*F*_1,22=_43.776, *p*<0.001), but no main effect of drug or interaction. Participants were more likely to hit on a win trial (mean=0.612, SD=0.079) compared to a neutral trial (mean=0.437, SD=0.072).

### MID fMRI results

Movement did not exceed 3 mm (our voxel size) in any direction for any of the participants. Mean and maximum movements were: *x*: mean=0.15 mm (SD=0.50 mm), max=0.50 mm; *y*: mean=0.19 mm (SD=0.12), max=0.50 mm; *z*: mean=0.34 mm (SD=0.32 mm), max=2.00. Therefore we did not exclude any participants for excess movement.

#### Whole brain analyses

##### Effects of task (Table 2; Figures 2 and 3)

**Figure 2.**
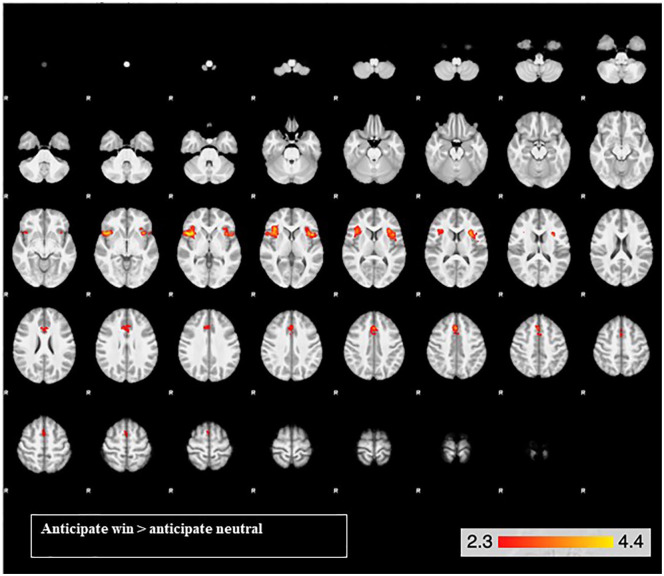
Brain activation for the reward anticipation contrast across both drug conditions in the bilateral insula cortex and the paracingulate gyrus. From the top left, slice images progress upward, on a ventral dorsal trajectory. The colours represent *z* values.

For the reward anticipation contrast, there was activation in three clusters, with peak activations in the insula bilaterally and the right paracingulate gyrus (Table 2). The right and left insula clusters extended into the right and left frontal operculum cortex, inferior frontal gyrus and orbitofrontal cortex. The paracingulate gyrus extended into the anterior cingulate gyrus, supplementary motor cortex and superior frontal gyrus (Figure 2).

**Table 2.** Activations for the reward anticipation (anticipate-win>anticipate-neutral) and reward feedback (feedback-win-hit>feedback-neutral-hit) contrast across both drug conditions. The table shows, for each cluster: the brain regions; cluster-corrected *p* values for each cluster; *k* (the size of each cluster, in terms of number of voxels); *z* value for the peak in the cluster; and the co-ordinates for the centre of gravity (COG) in Montreal Neurological Institute (MNI) space.

Region	*p* (FWE-corrected)	*k*	*z*	COG co-ordinates in cluster (MNI, mm)		
				*x*	y	z
**Reward anticipation**						
Right insula	0.003	801	4.42	41.1	18.2	2.4
Left insula	0.008	702	3.89	–38	14.5	15.2
Paracingulate gyrus	0.013	642	3.65	1.9	19.0	40.7
**Reward feedback**						
Right occipital fusiform gyrus	<0.001	79376	6.62	4.6	–52.3	3.5
Left precentral gyrus	<0.001	2073	4.68	–40.6	6.8	34.4

**Figure 3.**
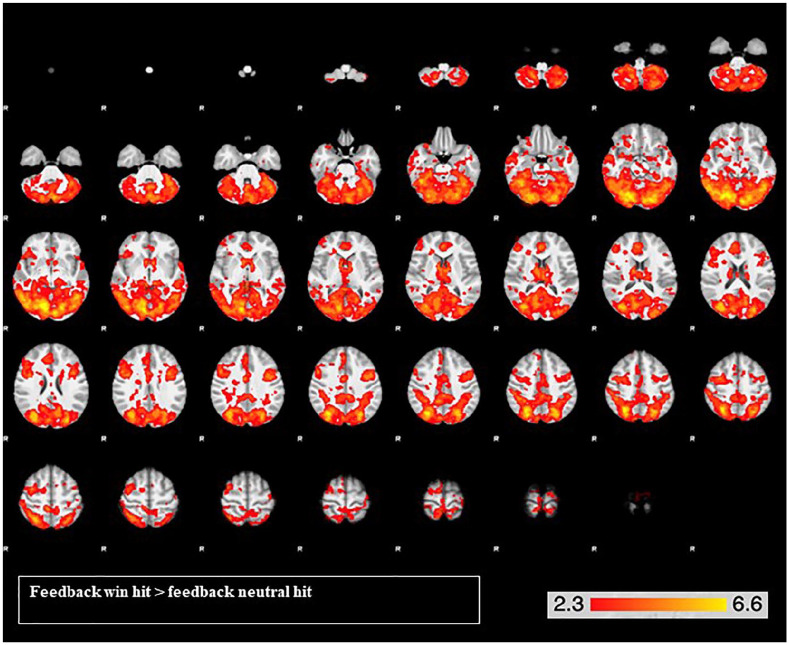
Widespread brain activation for the reward feedback contrast across both drug conditions, with peak activations in the left occipital fusiform gyrus and the right precentral gyrus. From the top left, slice images progress upward, on a ventral-dorsal trajectory. The colours represent *z* values.

For the reward feedback contrast, there was very widespread activation in two large clusters: one more posterior and one more anterior (Table 2; Figure 3). The posterior had a peak activation in the left occipital fusiform gyrus and extended into the bilateral cerebellum, intracalcarine gyrus, lingual gyrus, precuneus, inferior and middle temporal cortex, anterior and posterior lateral occipital gyrus, postcentral gyrus, posterior supramarginal gyrus and hippocampus, amongst others. The anterior cluster had a peak activation in the left precentral gyrus and extended into the bilateral anterior cingulate cortex, paracingulate gyrus, superior and middle frontal gyrus, frontal pole, precentral gyrus, frontal medial cortex and frontal operculum, amongst others. Activity was also observed in bilateral caudate, accumbens, thalamus and pallidum.

##### Effects of the drug

No significant clusters were found for CBD>PBO or PBO>CBD for either reward anticipation or feedback.

#### ROI analyses

##### Effects of task (Table 3)

**Table 3.** Effect of monetary incentive delay (MID) task (reward anticipation and feedback) on brain activity in our regions of interest (ROIs), showing *t*-statistics and *p*-values. Degrees of freedom=23 for each test. Alpha value was reduced to 0.006 to account for multiple tests.

Region	*t*	*p*
Reward anticipation		
L medial frontal gyrus	0.962	0.347
**R insula**	**3.87**	**0.001** ^a^
R NAcc	–1.25	0.225
L NAcc	–0.06	0.950
R thalamus	0.11	0.915
L thalamus I	–1.68	0.108
L thalamus II	–2.03	0.055
L culmen	2.05	0.052
Reward feedback		
R subcallosal gyrus	2.22	0.037
**L parahippocampal gyrus**	**3.31**	**0.003** ^a^
**R parahippocampal gyrus**	**3.38**	**0.003** ^a^
**R caudate**	**3.46**	**0.002** ^a^
R NAcc	2.28	0.033
**L NAcc**	**4.02**	**0.001** ^a^
L amygdala	2.22	0.037

L: left; NAcc: nucleus accumbens; R: right.

a Significant results are highlighted in bold.

For reward anticipation, only the right insula was significantly activated (*t*_22=_3.87, *p*=0.001) during reward anticipation.

For reward feedback, the left (*t*_22=_3.31, *p*=0.003) and right (*t*_22=_3.38, *p*=0.003) parahippocampal gyri, right caudate (*t*_22=_3.46, *p*=0.002) and left NAcc (*t*_22=_4.02, *p*=0.001) were significantly activated during reward feedback.

##### Effects of drug (Table 4)

**Table 4.** Effect of cannabidiol (CBD) on brain activity during reward anticipation and feedback in our regions of interest (ROIs), showing *t*-statistics, *p*-values and Bayes factors. Degrees of freedom=23 for each test. Alpha value was reduced to 0.006 to account for multiple tests. A Bayes factor of >3 is taken in support of the null.

Region	*t*	*p*	Bayes factor
Reward anticipate			
L medial frontal gyrus	1.04	0.309	3.75
R insula	0.232	0.819	6.09
R NAcc	–0.639	0.530	5.14
L NAcc	1.34	0.194	2.71
R thalamus	0.203	0.841	6.13
L thalamus I	0.543	0.592	5.43
L thalamus II	0.404	0.690	5.78
L culmen	–0.972	0.342	3.99
Reward feedback			
R subcallosal gyrus	–0.475	0.640	5.61
L parahippocampal gyrus	0.842	0.409	4.46
R parahippocampal gyrus	–0.543	0.593	5.43
R caudate	0.116	0.909	6.21
R NAcc	–0.223	0.826	6.10
L NAcc	–0.952	0.351	4.07
L amygdala	–0.158	0.876	6.18

L: left; NAcc: nucleus accumbens; R: right.

CBD did not differ from PBO in all of the ROIs during reward anticipation (*p*s>0.1). Furthermore, all but one of the ROIs had a Bayes factor >3, in favour of there being no difference between drug conditions.

CBD did not differ from PBO in all of the ROIs during reward feedback (*p*s>0.3). Furthermore, all the ROIs had Bayes factors >3, in favour of there being no difference between drug conditions.

### Correlations

There were no significant correlations between plasma CBD levels and activation in any of the ROIs during anticipation or feedback.

## Discussion

We hypothesised that brain activity would be greater during reward anticipation and feedback following 600 mg of oral CBD compared to PBO. However, this was not the case. We found no evidence that CBD affects the brain’s response to reward anticipation or feedback. Furthermore, in pre-specified reward-related brain regions (Knutson and Greer, 2008), using Bayesian analyses, we found support for there being no difference in neural activity between CBD and PBO. Overall, we found no support for CBD affecting the neural correlates of reward anticipation and feedback or behavioural measures of motivation for reward in healthy volunteers.

Across both drug conditions, in the whole brain, our MID task elicited reward anticipation activation in the bilateral insula and paracingulate gyrus, extending into the inferior frontal gyri and orbitofrontal cortex. In our ROI analysis, the right insula was significantly activated during reward anticipation. Reward feedback elicited extensive activity across anterior and posterior parts of the brain, including a range of reward-related brain regions. In our ROI analysis, the right caudate, left NAcc and bilateral parahippocampal gyri were activated during reward feedback. These analyses demonstrate that anticipation and feedback of reward produced activity in several expected brain regions. Further support that the task functioned adequately is that both RT and hit rate were significantly affected by trial type, such that participants were faster and more likely to successfully hit the target on win trials compared to neutral trials. Importantly, our plasma results demonstrate that the 600 mg oral dose of CBD was absorbed.

In terms of behavioural outcomes, CBD led to longer RTs compared to PBO overall. However, there was no interaction between drug and trial-type; CBD did not reduce RTs more for win trials than it did for neutral trials. Hence CBD did not affect our behavioural measure of motivation for reward; it simply increased RT, in general (i.e. comparably for both trial-types). This is somewhat surprising given previous research has not found CBD to affect reaction speed in general (Belgrave et al., 1979; Fusar-Poli et al., 2009; Hindocha et al., 2018).

Despite some existing evidence that CBD can impact reward function, we found null results for its effects on the neural correlates of reward anticipation and feedback. This absence of impact on reward circuitry, may contribute to the lack of reinforcing and abuse potential of CBD (Haney et al., 2016). To our knowledge, no previous study has examined the effects of CBD alone on brain activity associated with reward processing or motivation for reward. Previous studies have often investigated how inhaled CBD moderates THC’s effects (Freeman et al., 2018; Lawn et al., 2016), which may have contributed to the discrepancy. Moreover, other studies have explored more complex components of reward function, including attentional bias toward drug pictures (Hindocha et al., 2018; Morgan et al., 2010). Other components of reward processing, including reward learning and subjective pleasure could also still be sensitive to a 600 mg dose of oral CBD. CBD’s acute effects on human behaviour and subjective experience are seemingly complicated and enigmatic (Bergamaschi et al., 2011; Fusar-Poli et al., 2009; Haney et al., 2016; Morgan et al., 2010). The same may well be true with regards to CBD’s impacts on reward processing.

Furthermore, long-term daily administration of CBD, as delivered in clinical trials (Freeman et al., 2020; Leweke et al., 2012; McGuire et al., 2018), could produce different effects on the neural correlates of reward anticipation and feedback. We only delivered a single oral 600 mg dose in healthy volunteers. CBD likely has complex, variable dose-response functions on diverse psychological outcomes (Zuardi et al., 2017). Nevertheless, experimental medicine approaches, such as this one, are needed to efficiently examine the acute effects of potentially therapeutic drugs in human models of psychiatric targets, where clinical trials are costly and protracted. Future research into CBD’s effects on reward processing should expand the reward components assessed and utilise different doses. It should also examine consequences of repeated, long-term administration, which may allow for CBD levels to build up in the body and have greater impacts on receptor expression and endocannabinoid levels.

The present results leave open the intriguing possibility that CBD may only exert an effect on reward networks that have already been perturbed, for example in people with a drug addiction. CBD administration has been shown to modulate reward-related behaviours in animals when addiction is being modelled (Katsidoni et al., 2013; Parker et al., 2004; Ren et al., 2009; Schier et al., 2014; Viudez-Martínez et al., 2018). Moreover, behavioural evidence from human studies suggests that CBD can reduce the salience of drug-related cues in those with cannabis (Morgan et al., 2010) and nicotine (Hindocha et al., 2018) dependencies, and reduce drug cue-induced cravings in those addicted to heroin (Hurd et al., 2019). Additionally, a four-week treatment of CBD dose-dependently decreased cannabis use in a clinical trial of people with cannabis use disorder (Freeman et al., 2020). In all of these studies, CBD attenuated atypical reward-related behaviours conferred by addiction, suggesting a restorative effect. Therefore, the null findings reported in the present study could have resulted from our sample of healthy volunteers. Future neuroimaging research should therefore administer CBD to participants thought to have perturbed reward systems, including those with addiction.

The reward system is thought to be critically involved in the emergence and/or maintenance of a variety of psychiatric disorders, including depression (Nestler and Carlezon, 2006; Whitton et al., 2016), schizophrenia (Kapur et al., 2005; Whitton et al., 2016) and addiction (Berridge and Robinson, 2016; Goldstein and Volkow, 2011). If it emerges that CBD does have accepted therapeutic effects in these domains, further research will be needed to understand whether or not the mechanism is related to reward circuitry. Moreover, an improved understanding of CBD’s pharmacological actions and their relative importance in treating reward-related psychological symptoms will be important in the development of cannabinoid-based psychiatric medicines. One possible avenue for future research would be to further understand and capitalise on CBD’s agonism of the serotonin-1a receptor (Russo et al., 2005), in order to potentially disrupt addition and depressive symptoms.

### Strengths and limitations

Our study has a number of strengths. First and foremost, it was a double-blind, placebo-controlled experiment addressing a novel and important research question. Second, we utilised a well-validated fMRI task which elicited activity in many expected brain regions and appropriately affected behavioural performance. Third, CBD was absorbed into the bloodstream. Fourth, we conducted Bayesian analyses to provide support for null findings.

However, there are some limitations. Despite stimulating activity in many expected brain regions, the MID failed to produce anticipatory activation in the striatum, which is the region most commonly found to respond in this stage of the task (Oldham et al., 2018). Thus, CBD could theoretically affect striatal activity (Bhattacharyya et al., 2010) and we may have failed to detect it here. Finally, although CBD was absorbed relative to PBO, our plasma levels were lower than that seen in previous oral CBD studies (Haney et al., 2016; Millar et al., 2018). This may have been caused by our fasting participants, as a large, high-fat meal eaten before CBD administration can augment bioavailability four-fold (Taylor et al., 2018). Therefore, we cannot exclude the possibility that if greater quantities of CBD had been absorbed, we may have observed different results. We also do not know whether 600 mg is the optimal dose to manipulate reward processing, especially given CBD’s potentially inverted U-shaped dose-response curve (Zuardi et al., 2017). Additionally, we did not control or account for female participants being in different stages of their menstrual cycle, which can affect psychopharmacological phenomena (Bolea-Alamanac et al., 2018).

## Conclusion

To conclude, in healthy volunteers, a single, oral 600 mg dose of CBD did not affect the neural correlates of reward anticipation and feedback, or behavioural measures of motivation for reward.
